# Sampling Extension, Chronic Infiltrates, and Eosinophils: Support for the Evaluation of Histological Healing in Inflammatory Bowel Disease with Endoscopic Remission

**DOI:** 10.3390/diagnostics16050739

**Published:** 2026-03-02

**Authors:** Gabriella Canavese, Enrico Costantino Falco, Davide Giuseppe Ribaldone

**Affiliations:** 1 Department of Pathology, Città Della Salute e Della Scienza di Torino, 10126 Turin, Italy; 2 Department of Medical Sciences, University of Turin, 10126 Turin, Italy; davidegiuseppe.ribaldone@unito.it

**Keywords:** histological healing, inflammatory bowel disease, residual disease

## Abstract

**Background/Objectives:** Histological healing, primarily assessed by the absence of neutrophils in mucosal biopsies, is increasingly used to evaluate treatment efficacy in inflammatory bowel disease (IBD) and may identify residual inflammation despite endoscopic mucosal healing. We aimed to quantify histological parameters commonly linked to active disease in patients with endoscopic healing and to explore their association with neutrophil-defined histologic activity in endoscopically healed mucosa. **Methods:** We assessed 371 colonoscopies from IBD patients with an endoscopic report of mucosal healing at a reference center. For each procedure, we recorded the number of biopsy samples obtained and histologic features according to ECCO consensus/position statements, including neutrophil infiltration, lymphoplasmacytic infiltrate, eosinophil infiltrate, and mucosal lesions. **Results:** Histologic activity was found in 21/98 (21.4%) procedures with one to three biopsy samples and in 89/273 (32.6%) procedures with more than three samples (*p* = 0.04). Neutrophils were absent in 207/212 (97.6%) procedures with normal lymphoplasmacytic infiltrate versus 55/159 (34.6%) with increased lymphoplasmacytic infiltrate (*p* < 0.00001). Eosinophils were below cut-off values in 122/168 (72.6%) procedures with normal lymphoplasmacytic infiltrate versus 90/203 (44.3%) with increased lymphoplasmacytic infiltrate (*p* < 0.00001). Eosinophils were below cut-off in 148/168 (88.1%) procedures without neutrophils and in 114/203 (56.2%) with neutrophils in the lamina propria (*p* < 0.00001). **Conclusions:** In IBD patients with endoscopic healing, the extent of biopsy sampling is associated with the detection of histologic activity. Lymphoplasmacytic and eosinophil infiltrates are strongly associated with neutrophil presence and are associated with neutrophil-defined activity and may serve as supportive indicators prompting closer pathological assessment in endoscopically healed mucosa.

## 1. Introduction

Inflammatory bowel disease (IBD) is a long-standing debilitating disease with in-creasing rates of global incidence and prevalence [1]; assessing disease status over time by means of clinical evaluation, endoscopy, and histology is pivotal in the therapeutic management of this pathology. In recent decades, gastrointestinal pathologists have developed a series of evaluation systems to quantify disease severity in histological samples. These scoring systems are designed on the basis of quantitative evaluations of the most significant tissue changes related to the disease (crypt distortion, discontinuity of the mucosal surface, lymphoplasmacytic (chronic) infiltrate, eosinophil infiltrate, and neutrophil infiltrate) and evolved from multiparametric scores (Geboes Score [2]) to one, two, or three-tiered simplified systems, integrated with an algorithmic criterium of histologic lesion classification (Nancy, Robarts [3,4]).

These scoring systems were extensively applied, and all of them demonstrated a strong correlation with clinical courses and an independent prognostic value compared with endoscopic scores [5,6,7]. Furthermore, histological activity was found in a significant proportion (30–50%) of endoscopies with no sign of disease [6,7].

However, some limitations were revealed by some authors [8,9,10,11]: as expected, these evaluation systems have been applied in academic contexts, by experienced researchers, and under optimal conditions of collaboration with other specialists and with ideal specimen quality; in contrast, the constant increase in the number of patients treated for IBD highlights the difficulties in employing the histologic scores for mucosa healing in the real-life practice of pathology laboratories. Subjectivity and poor agreement in assessment represent major limitations in the standardization of histological healing assessment.

In recent years, the therapeutic efficacy in patients with IBD has been improved by the introduction of biological drugs, a new category of medication that comprises a series of monoclonal antibodies directed to the main molecular modulators of the inflammatory response in bowel mucosa. Treatment with biologics leads, in a percentage of patients, to rapid and widespread involution of the inflammatory response, with a possible complete restitutio ad integrum of the mucosa (deep remission, disease clearance). Until now, endoscopic healing, defined as MES 0 in UC and the absence of ulcers in CD (e.g., SES-CD ≤ 2), has been used as a validated index of disease remission, but recently STRIDE II consensus on treatment targets has considered histological healing, defined as the absence of active inflammation on endoscopic mucosal biopsies, as a reliable and sensitive independent predictor of complete response to treatment, by virtue of its ability to detect active disease in patients with endoscopic healing [11].

Therefore, histological mucosal healing, defined as the disappearance of the histological signs of disease activity, has been proposed as a future treatment target in patients with IBD (in particular, in ulcerative colitis, UC); hence, scientific research in the field and medical societies projects moved towards the codification of increasingly precise, sensible, and easy-to-use criteria of evaluation. New scoring systems such as the PICASSO histological score and Villanacci score, supported by new techniques such as digital scan image analysis and AI tools, have adopted neutrophils as a unique parameter of active disease [12,13,14,15].

Some issues must be considered in assessing disease status in the post-STRIDE era. First, it must be considered that we still lack a univocal and shared definition of complete histological remission: the absence of activity has been defined as (1) the absence of intraepithelial neutrophils, (2) the absence of neutrophils in lamina propria, and (3) the absence of the characteristic infiltrate of plasma cells and eosinophils in lamina propria. The different definitions of “healing” create a bias when translated in the available score systems: for instance, the Nancy score encompasses mild chronic infiltrate and the absence of chronic infiltrate in a unique score. The added value of histological mucosal healing in IBD management is observed in patients without endoscopic lesions, i.e., biopsies showing minimal histological activity characterized by the absence of granulocytes. In any case, the above-described limitations in the evaluation of activity scores in routine practice must be considered.

To contribute to the debate about the above-described topics, we performed a retrospective investigation on a consecutive series of sampling performed in a reference center involving IBD patients with endoscopic healing, investigating the distribution of parameters commonly considered in the assessment of disease activity.

## 2. Materials and Methods

The present study is based on a cohort of 371 cases of endoscopic sampling from the files of the institute of Pathology of the University of Turin, Italy.

Patients undergoing follow-up in the gastroenterology department of our institution (“A.O.U. Città della Salute e della Scienza di Torino”) and with an endoscopic report of mucosal healing from endoscopies of the same institution were selected for this study.

Inclusion criteria. Consecutive follow-up colonoscopic biopsy cases from patients with a diagnosis of IBD (UC, Crohn’s disease—CD, or IBD—unclassified—IBD-U) followed at our institution and with an endoscopic report of mucosal healing from procedures performed at the same center. Multiple endoscopies of the same patient were not included in the study.

Endoscopic mucosal healing was defined as MES 0 in UC and, in CD, as the absence of ulcerations in ileocolonoscopy. When an endoscopic activity score was reported, endoscopic healing was operationalized as SES-CD ≤ 2, a conservative threshold consistent with STRIDE-II recommendations (SES-CD < 3 and/or absence of ulcerations) and chosen to minimize the risk of including clinically relevant residual mucosal inflammation [12].

Exclusion criteria. Non-IBD diagnoses or the absence of an endoscopic report documenting mucosal healing. No exclusions were made based on the number of biopsy samples or technical sampling difficulties. Cases lacking first-diagnosis histology at our center were retained when external histology reports were available.

Variables analyzed:

• Demographics/clinical: Age at colonoscopy; sex; IBD subtype (UC, CD, IBD-U); disease duration; disease extent/phenotype (Montreal classification for CD and extent for UC, when available); smoking status; prior IBD-related surgery (yes/no); and ongoing IBD therapy at the time of colonoscopy (5-ASA, systemic steroids, immunomodulators, biologics, and small molecules; categorized by class). Where available, C-reactive protein (CRP) and fecal calprotectin values obtained within 30 days before/after colonoscopy were recorded.
• Endoscopy/sampling: The number of biopsies per colonoscopy; notes on technical difficulties; activity assessed for each sampled segment.
• Histopathology (per ECCO/SIAPEC guidance): Intraepithelial/lamina propria neutrophils (absent, sporadic, cryptitis, crypt abscesses); lymphoplasmacytic (chronic) infiltrate (semi-quantitative grading); eosinophils (within normal range vs. increased, considering segmental physiological gradients); surface epithelial discontinuities (absent, erosions, ulcers); chronic damage (crypt atrophy/fibrosis); crypt distortion (present/absent).

The collected histopathological reports were designed following the recommendations of the Gastroenterology section (GIPAD) of the Italian Society of Pathology and Cytology (SIAPEC) [16].

The morphological parameters were evaluated considering the ECCO consensus and position statement [17,18,19,20,21]. Along with the cited statement, the following scoring criteria were adopted for the present study:

• Inflammatory activity is traditionally represented by the presence of neutrophils in mucosal structures. In our study, activity was classified as minimal (scattered neutrophils in the lamina propria or/and the crypt epithelium and/or superficial epithelium), mild (multiple clusters of neutrophils the crypt epithelium and/or superficial epithelium), moderate (diffuse cluster of neutrophils the crypt epithelium and/or superficial epithelium), or severe (same as moderate, with crypt abscesses).
• Chronic inflammation was defined as an increased mononuclear infiltrate (lymphocytes and mature plasma cells, and usually less represented populations such as histiocytes and monocytes) in the lamina propria, as reported in routine pathology. Because no universally accepted grading system is routinely applied in clinical reports, chronic inflammatory infiltrate was categorized semi-quantitatively as mild, moderate, or severe based on report wording and overall cellular density: mild = slightly increased above background with dispersed cells; moderate = clearly increased cellularity without confluent packing; severe = dense, closely packed mononuclear infiltrate.
• There is no widely accepted definition of a significant increase in colorectal mucosal eosinophils in IBD (ECCO Position 4.2 [19]), and the evaluation is complicated by the uneven distribution along the colonic tract (decreasing from the ileum to the rectum). In the present study, we used the following cut-offs: about 40 in the ileum—ascending colon to drop to approximately 20× HPF in the sigma rectum, used in reference papers [22].
• Surface epithelial discontinuities were distinguished in lesions with (ulcers) or without (erosions) evidence of granulation tissue.
• The presence or absence of chronic damage (crypt atrophy, fibrosis).
• The occurrence of crypt distortion was defined as present or absent.

All analyses and descriptive characteristics were summarized at the procedure level (each eligible colonoscopy), consistent with the unit of analysis for histologic assessments.

Longitudinal clinical outcomes (e.g., relapse, hospitalization, treatment escalation, surgery) were not systematically retrievable from the medical records for this retrospective cohort and were not analyzed.

Activity was evaluated and reported for every segment of endoscopic sampling.

Primary outcome. The proportion of cases with histological activity (i.e., presence of neutrophils) among patients with endoscopic healing and its association with sampling extension (≤3 vs. >3 samples).

Secondary outcomes. (i) The association between neutrophil infiltration and the density of the lymphoplasmacytic infiltrate; (ii) the association between neutrophils and eosinophil counts (below vs. above cut-off); and (iii) the descriptive distribution of other histological parameters listed above.

### Statistical Methods

Categorical variables were summarized as counts (percentages) and compared using the χ^2^ test or Fisher’s exact test where appropriate; continuous variables were summarized as mean (SD) or median (IQR) as applicable. All tests were two-sided with α = 0.05. The association between sampling extension (≤3 vs. >3 samples) and the presence of histologic activity was assessed with contingency testing as above. *p*-values are reported where relevant.

Multiplicity. The study was designed around a single prespecified primary comparison (sampling extent ≤3 vs. >3 sampled segments and detection of histologic activity). Accordingly, no adjustment for multiple comparisons was applied to the primary analysis. All additional analyses (including subtype-stratified comparisons and ancillary histologic associations) were considered exploratory; *p*-values for these analyses are therefore reported as nominal/descriptive. For transparency, false discovery rate (Benjamini–Hochberg)-adjusted *p*-values are provided for the exploratory subgroup comparisons (Table S1).

Multivariable analysis. To account for potential confounding, we fitted a multivariable logistic regression model with histologic activity (presence of neutrophils; yes/no) as the dependent variable. The main exposure was sampling extent (≤3 vs. >3 sampled bowel segments). Prespecified covariates included IBD subtype (UC, CD, IBD-U), disease duration (years), and ongoing IBD therapy at the time of colonoscopy (coded by medication class). Adjusted odds ratios (aORs) with 95% confidence intervals (CIs) are reported. Analyses were conducted as complete case for variables included in the model.

The primary analysis pooled UC, CD, and IBD—unclassified procedures because the exposure of interest (sampling extent ≤3 vs. >3 colonic segments) and the primary endpoint (histologic activity defined by neutrophils) are defined consistently across subtypes. Disease subtype was addressed by (i) prespecified subtype-stratified analyses and (ii) adjustment for subtype in multivariable regression. Heterogeneity of the sampling effect across subtypes was evaluated by testing for the equality of odds ratios across strata.

Given the retrospective, exploratory design of this single-center study, no formal a priori sample size calculation was performed. The sample size was determined by feasibility and corresponds to all consecutive colonoscopic biopsy procedures that met the predefined inclusion criteria and were available in the institutional pathology archives during the study period, yielding 371 colonoscopies. With this sample size, the precision for estimating the prevalence of histologic activity is approximately ±5% using 95% confidence intervals for proportions in the range observed in similar cohorts.

## 3. Results

The mean age of the cohort was 52.3 (range 10–91). In 36 cases (9.7%), the histological material relating to the first diagnosis was not available in our center, and the evaluation was based on reports from other laboratories. The cohort comprised 227 UC (61.2%), 104 CD (28.0%), and 40 IBD-U (10.8%) cases. Low-grade dysplastic lesions were found in eight cases (2.2%).

Baseline demographic and clinical characteristics of the cohort are summarized in Table 1, including sex distribution, IBD subtype, disease duration, extent/phenotype (when available), prior surgery, and ongoing IBD therapy at the time of colonoscopy, together with routine inflammatory biomarkers around the procedure when available.

The distribution of the segments sampled in the endoscopies included in this study is summarized in Table 2.

The mean number of samples for each colonoscopy in the series was 4.4. In 98/371 cases (26.4%), ≤3 biopsies were obtained, i.e., below the recommended sampling extent for follow-up colonoscopies. In six cases (1.6%, of which two were endoscopies with ≤3 samplings), technical difficulties in sampling (previous resections, stenosis, etc.) were re-ported.

As expected, the sampling distribution is different in the two types of disease (Crohn’s and UC), and these data also demonstrate unsatisfactory sampling: in 27/104 cases (25.9%) of Crohn’s cases, ileal sampling was not available, and, considering UC patients, in 59/227 cases (25.9%) and in 65/227 cases (28.6%), respectively, sampling of the descending and sigmoid segments was not available.

When comparing procedures with limited sampling (1–3 bowel segments) versus those with more extensive sampling (>3 segments), histologic activity (presence of neutrophils) was detected in 21/98 (21.4%) versus 89/273 (32.6%) procedures, respectively (OR 1.77, 95% CI 1.03–3.06; Fisher’s exact *p* = 0.040). In the multivariable model adjusting for IBD subtype, disease duration, and ongoing therapy class, sampling >3 segments remained associated with higher odds of detecting histologic activity (aOR 1.82 (95% CI 1.05–3.16), *p* = 0.034). Full model results are shown in Table S1.

Data on the scores calculated for all cited histological parameters, as described in the Materials and Methods Section, are summarized in Table 3.

Neutrophils were not detectable in 207 of 212 (97.6%) cases with normal density of lymphoplasmacytic infiltrate in the mucosal lamina propria and in 55 of 159 cases with increased lymphoplasmacytic infiltrate (34.6%) (*p* value < 0.00001). In six cases (1.6%), the activity was limited to inflammatory pseudo polyps.

**Figure 1:**
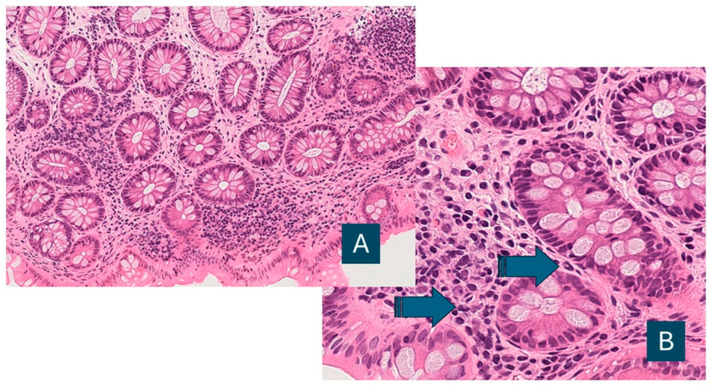
Case 292, sample D (**A**): neutrophil: absent; lymphoplasmacytic infiltrate: slight increase; eosinophils: in normal range (hem eos, OM 100×). (**B**) Note elements with suggestive morphology for neutrophils (arrows) in lamina propria.

**Figure 2:**
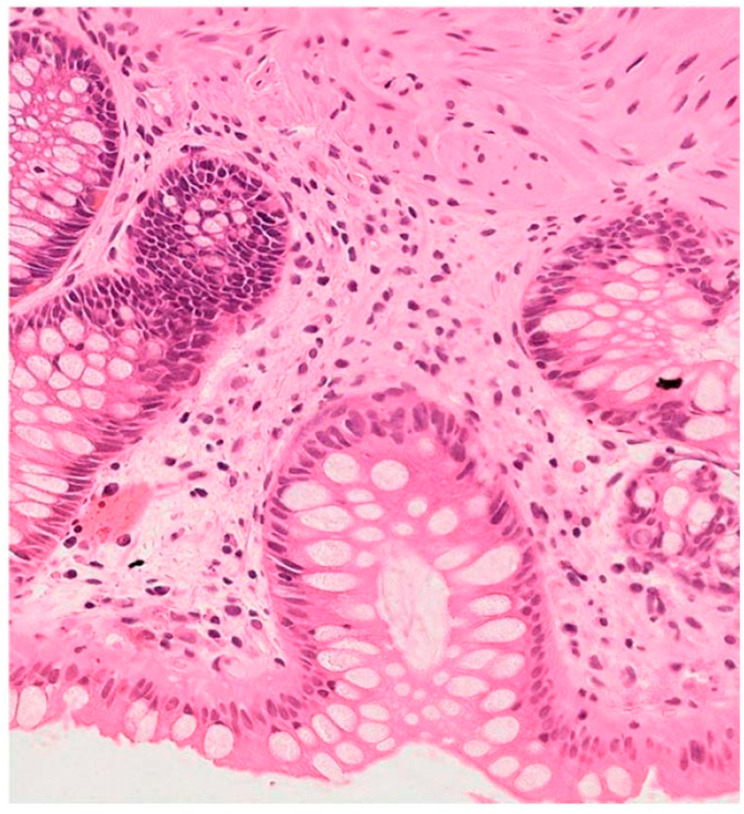
Case 292, sample E: (hem eos, OM 100×) sample with lymphoplasmacytic infiltrate in normal range.

**Figure 3:**
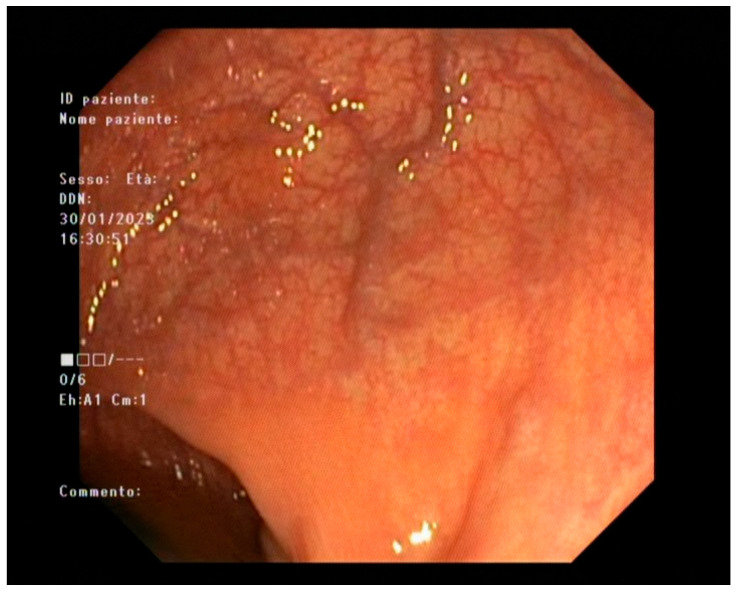
Case 292 endoscopy (Mayo 0).

Counts of eosinophil granulocytes in the lamina propria were below the cut-off values in 122 of 168 (72.6%) cases with lymphoplasmacytic infiltrate in the normal range and in 90 of 203 cases (44.3%) with increased lymphoplasmacytic infiltrate (*p* < 0.00001). Furthermore, eosinophils were below cut-off values in 148 of 168 (88.1%) cases with no evident neutrophil infiltrate and in 114 of 203 (56.2%) cases with neutrophils in the lamina propria (*p* < 0.00001) (Figure 1, Figure 2 and Figure 3).

### Subgroup Analyses by Disease Subtype

When stratified by IBD subtype, histologic activity was detected in 73/227 (32.2%) UC procedures, 29/104 (27.9%) CD procedures, and 8/40 (20.0%) IBD-U procedures. Within UC, histologic activity was detected in 13/58 (22.4%) procedures with 1–3 sampled segments versus 60/169 (35.5%) with >3 sampled segments (nominal *p* = 0.07). Within CD, the corresponding proportions were 6/29 (20.7%) versus 23/75 (30.7%) (nominal *p* = 0.31). In IBD-U, the corresponding proportions were 2/11 (18.2%) versus 6/29 (20.7%), and estimates are provided descriptively given the limited subgroup size (Table S2). No evidence of heterogeneity of the sampling effect across subtypes was observed (test for equal odds ratios across strata: *p* = 0.88).

## 4. Discussion

The clinical course of IBD has now radically improved due to the introduction of biological drugs in therapeutic protocols. Therefore, measurement of disease severity is becoming essential in clinical practice to check the effectiveness of the therapy. Evidence of residual disease activity in tissue samples from a significant number of patients with complete remission of endoscopic mucosal lesions (endoscopic healing) has been proven in many studies [6,7,8], and as reported in a recent consensus (STRIDE II, 2021) [11], histology has gained an emerging role as a sensible technique for defining the complete remission of the disease and was proposed as a possible evolution of endoscopic healing, the technique previously used for the assessment of disease healing.

Therefore, careful identification of neutrophils in mucosal biopsies has gained relevance for characterizing residual inflammatory activity in endoscopically healed mucosa, although the present study was not designed to evaluate treatment outcomes or to support treatment decision-making [23].

In our retrospective survey, data on clinics, endoscopic sampling, and histological parameters (listed and evaluated in the reports according to the national and European guidelines) were collected from a series of colonoscopy samples from patients with endoscopic healing. We focused our analysis on chronic inflammatory cell infiltrates, eosinophils, and neutrophils—the leukocyte populations most closely associated with active disease—because (1) crypt distortions are not directly related to the active phase of the disease and (2) mucosal discontinuities (erosions/ulcerations) are poorly represented in cases with endoscopic healing (0.5% of cases with ulcerations in our cohort) and their assessment is somewhat subjective.

Histological activity was detected in approximately one-third of the cases of endoscopic healing assessed in this study, demonstrating an additional contribution of morphology in identifying activity in a series of routine endoscopies from a single reference center. This evidence confirms data from previous studies [6] and meta-analyses that revealed an independent prognostic value for this exam, positioning this technique as the ideal candidate for monitoring the efficacy of treatment, as hypothesized by the STRIDE II consensus [12].

Our findings revealed further interesting evidence. First, sampling is still suboptimal in our series: the number of sampled segments is ≤3 in 26.4% of cases. If we compare this subgroup with the cases with more than three samples, the sampling topographic extension is statistically related to the evidence of neutrophils in samples from these endoscopies. Moreover, sampling of significant segments in Crohn’s (ileum) and UC (descending and sigmoid) was not available in about one-third of endoscopies. These acquisitions confirm the need for sampling criteria standardization in endoscopy routine practice to improve the consistency and comparability of histologic assessment across follow-up procedures, as already highlighted in a study of our group [24], and suggest that compliance with the guidelines could enhance sensibility in detecting residual active disease in histology, taking the ECCO indications for IBD in first-approach endoscopies as a reference (a minimum of two biopsies from at least five sites around the colon [including the rectum] and the ileum should be obtained for a reliable diagnosis) [17,18]. Importantly, this association persisted after adjustment for disease subtype, disease duration, and ongoing therapy (aOR 1.82, 95% CI 1.05–3.16; *p* = 0.034), supporting the robustness of the finding, although residual confounding related to non-standardized biopsy strategy cannot be excluded. The recent ‘Inflammatory Bowel Disease—Distribution, Chronicity, Activity [IBD-DCA] Score’ is an interesting example of a histological healing evaluation system comprising chronic infiltrate and the distribution of inflammation [25].

Although UC, CD, and IBD—unclassified differ in histologic behavior and remission definitions, pooling was appropriate for our primary methodological question (sampling adequacy), and the association between more extensive sampling and higher detection of histologic activity showed a consistent direction across subtypes with no evidence of effect heterogeneity (Table S2); nevertheless, subgroup inferences—particularly for IBD—unclassified—remain exploratory due to limited numbers.

On the other hand, this retrospective study demonstrated a strong correlation between the occurrence of neutrophils and the detection of other white-series elements of the inflammatory infiltrate (lymphocytes, plasmacytes, and eosinophils). In effect, the role of these cell populations has been recognized in the literature and they have been proposed as parameters in many disease activity scores, with their prognostic significance already proven. Moreover, studies on experimental models showed that a group of interleukins regulates the crosstalk between lymphocytes and neutrophils and between lymphocytes and eosinophils [26,27,28].

Given the retrospective design and reliance on routine pathology reporting, these associations should be interpreted as observational correlates rather than as evidence to modify histological remission definitions. Practically, when increased lymphoplasmacytic infiltrate and/or eosinophils are reported in biopsies from endoscopically healed mucosa, these features may serve as supportive indicators prompting closer pathological assessment (e.g., careful search for focal neutrophils and, when appropriate, additional levels), without implying specific treatment changes.

Regarding the limitations of our study, this was a retrospective, single-center analysis from a reference institution, which may limit generalizability. Data were extracted from routine histopathology reports compiled according to GIPAD/SIAPEC and ECCO guidance rather than by centralized blinded slide re-review, introducing potential information bias. Quantification of lymphoplasmacytic (chronic) infiltrate lacks fully standardized, reproducible cut-offs and can be affected by technical factors (e.g., section thickness, staining), limiting between-case comparability. Although eosinophils were graded acknowledging physiological proximal-to-distal variation, some misclassification is possible. Because multiple secondary and subgroup analyses were exploratory, and *p*-values for these comparisons are nominal, the possibility of inflated type I error cannot be excluded; therefore, these findings should be interpreted cautiously and confirmed in prospective studies with standardized biopsy protocols. Clinical outcome data during follow-up (e.g., relapse, hospitalization, need for treatment escalation or surgery) were not consistently available from the medical records and could not be analyzed; therefore, we could not correlate histologic findings with subsequent clinical outcomes. Finally, because UC procedures were included only if MES was 0, we could not assess whether the observed relationships would be similar using a broader endoscopic definition (MES 0–1). Definitions of endoscopic remission/healing in CD are heterogeneous across studies; however, our cohort was restricted to the absence of ulcerations and, when available, a stringent SES-CD threshold (≤2), which falls within STRIDE-II endoscopic healing criteria. Therefore, generalizability to patients with MES 1 endoscopic remission cannot be inferred from the present data.

Despite the crucial role of the evaluation of lymphoplasmacytic infiltrate density, a technical limit, as previously stated, is represented by the lack of shared and reproducible criteria of quantification. The cut-off distinguishing the normal amount of resident lymphoplasmacytic infiltrate in the lamina propria and a true inflammatory condition is often somewhat subjective and can be affected by section thickness or staining defects. Optimal technical processing, the adoption of shared visual scales for chronic infiltrate grading, and a coordinated evaluation of lymphoplasmacytic population and eosinophilic granulocytes could be helpful in conducting a more precise evaluation of mucosal healing. Considering that the evaluation of these parameters in routine practice is demanding for the pathologist and time-consuming for the laboratory, the use of artificial intelligence with digitized preparations could make a significant contribution in this field of diagnostics [29].

## 5. Conclusions

In conclusion, the primary methodological finding of our study is that biopsy sampling extent significantly influences the detection of histologic activity in endoscopically healed IBD. In addition, lymphoplasmacytic and eosinophil infiltrates were associated with neutrophil-defined activity: these features should be interpreted as supportive indicators that may prompt closer pathological scrutiny for focal activity rather than as components of remission criteria.

Prospective studies with standardized biopsy protocols and longitudinal outcome capture are needed to determine whether these observational associations have independent prognostic value.

## Figures and Tables

**Table 1:** Baseline demographic, clinical, and endoscopic characteristics of the study cohort with endoscopic mucosal healing (procedure level).

Variable	Overall
Eligible colonoscopies with biopsies, *n*	371
Age, mean (range), years	52.3 (10–91)
Sex, male, *n* (%)	198 (53.4)
IBD subtype, *n* (%)	UC: 227 (61.2); CD: 104 (28.0); IBD-U: 40 (10.8)
Disease duration, median (IQR), years	8.5 (4.2–14.3)
UC extent (E1/E2/E3), *n* (%)	42 (18.5)/89 (39.2)/96 (42.3)
CD location (L1/L2/L3), *n* (%)	31 (29.8)/28 (26.9)/45 (43.3)
CD behavior (B1/B2/B3), *n* (%)	58 (55.8)/32 (30.8)/14 (13.4)
Perianal disease, *n* (%)	17 (16.3)
Prior IBD-related surgery, *n* (%)	64 (17.3)
Ongoing IBD therapy at colonoscopy, *n* (%)	5-ASA: 189 (50.9); Immunomodulators: 98 (26.4); Biologics: 142 (38.3); Small molecules: 23 (6.2); Steroids: 12 (3.2)
CRP within ±30 days, median (IQR), mg/L	2.1 (0.8–4.6)
Fecal calprotectin within ±30 days, median (IQR), µg/g	78 (32–165)
Definition of endoscopic healing used for eligibility, *n* (%)	MES 0 (UC): 227 (61.2); absence of ulcerations (or SES-CD ≤ 2 when reported) (CD): 104 (28.0); IBD-U: 40 (10.8)

UC, ulcerative colitis; CD, Crohn’s disease; IBD-U, inflammatory bowel disease—unclassified. E1, proctitis; E2, left-sided colitis; E3, extensive colitis (Montreal classification for UC extent). L1, ileal; L2, colonic; L3, ileocolonic (Montreal classification for CD location). B1, non-stricturing non-penetrating; B2, stricturing; B3, penetrating (Montreal classification for CD behavior). 5-ASA, 5-aminosalicylic acid. CRP, C-reactive protein; IQR, interquartile range. MES, Mayo Endoscopic Subscore; SES-CD, Simple Endoscopic Score for Crohn’s Disease.

**Table 2:** Distribution of sampling in cases with CD and UC.

CD (*n* = 104—3 with Previous Surgery, 1 with Stenosis)					UC (*n* = 227)				
	sampled		not sampled			sampled		not sampled	
ileum	77	74.04%	27	25.96%	ileum	94	41.41%	133	58.59%
ileocecal valve	8	7.69%	96	92.31%	ileocecal valve	10	4.41%	217	95.59%
cecum	15	14.42%	89	85.58%	cecum	41	18.30%	183	81.70%
ascending	87	83.65%	17	16.35%	ascending	184	81.06%	43	18.94%
transverse	71	68.27%	33	31.73%	transverse	164	72.25%	63	27.75%
descending	79	75.96%	25	24.04%	descending	168	74.01%	59	25.99%
sigmoid	45	43.27%	59	56.73%	sigmoid	162	71.37%	65	28.63%
rectum	79	75.96%	25	24.04%	rectum	195	85.90%	32	14.10%
Sampled segments in endoscopies included in study									
n° of sampled segments	n° of cases	% of cases							
8 sampled segments	3	0.81%							
7 sampled segments	15	4.04%							
6 sampled segments	75	20.22%							
5 sampled segments	109	29.38%							
4 sampled segments	71	19.14%							
3 sampled segments	49	13.21%							
2 sampled segments	31	8.36%							
1 sampled segment	18	4.85%							

**Table 3:** Histopathological features in study cohort with endoscopic mucosal healing.

Crypt Distortion	Absent	Present		
	156 (42.1%)	215 (57.9%)		
mucosal discontinuities	absent	erosions	ulcers	
	336 (90.6%)	33 (8.9%)	2 (0.5%)	
eosinophil granulocytes count	under CO values	over CO values		
	168 (45.3%)	203 (54.7%)		
intraepithelial neutrophils	absent	sporadic	cryptitis	abscesses
	262 (70.6%)	61 (16.4%)	33 (8.9%)	15 (4.1%)
fibrosis/atrophy	absent	present		
	195 (52.6%)	176 (47.4%)		
lymphoplasmacytic infiltrate	absent	faint	moderate	n.a.
	211 (56.9%)	120 (32.3%)	39 (10.5%)	1 (0.3%)

CO: cut-off; n.a.: not available.

## Data Availability

The original contributions presented in this study are included in the article. Further inquiries can be directed to the corresponding author.

## Supplementary Materials

The following supporting information can be downloaded at https://www.mdpi.com/article/10.3390/diagnostics16050739/s1, Table S1: Multivariable logistic regression for histologic activity (presence of neutrophils): association with sampling extent adjusted for subtype, disease duration, and therapy. Table S2: Primary outcome stratified by IBD subtype: histologic activity and sampling extent (1–3 vs. >3 sampled segments).

## Abbreviations

The following abbreviations are used in this manuscript:

AI	Artificial intelligence
CD	Crohn’s disease
CO	cut-off
ECCO	European Crohn’s and Colitis
GIPAD	Italian Group of Gastrointestinal Pathologists
IBD	inflammatory bowel disease
IBD-DCA	Inflammatory Bowel Disease—Distribution, Chronicity, Activity
IBD-U	inflammatory bowel disease—unclassified
IL	interleukin
IQR	interquartile range
MES	Mayo endoscopic subscore
n.a.	not available
OM	original magnification
SD	standard deviation
SES-CD	Simple Endoscopic Score for Crohn’s Disease
SIAPeC-IAP	Società Italiana di Anatomia Patologica e Citopatologia Diagnostica (Italian Society of Anatomic Pathology and Diagnostic Cytology)
STRIDE	Selecting Therapeutic Targets in Inflammatory Bowel Disease
Th	T helper
Th2	T helper 2
Th17	T helper 17
UC	ulcerative colitis
