# Supplementary material for: Sampling Extension, Chronic Infiltrates, and Eosinophils: Support for the Evaluation of Histological Healing in Inflammatory Bowel Disease with Endoscopic Remission
Source: Diagnostics (Basel). 2026 Mar 2;16(5):739. doi: 10.3390/diagnostics16050739 (PMC12984903; doi:10.3390/diagnostics16050739)
Supplement: Supplementary file 1 [file diagnostics-16-00739-s001.zip › Table S1.pdf]

Table S1. Multivariable logistic regression for histologic activity (presence of neutrophils): association with sampling extent adjusted for subtype, disease duration, and therapy.

Predictor	aOR	95% CI	p-value
**Sampling extent**			
>3 segments (vs ≤3)	1.82	1.05–3.16	0.034
**IBD subtype**			
CD (vs UC)	0.78	0.46–1.32	0.35
IBD-U (vs UC)	0.54	0.23–1.27	0.16
**Disease duration**			
Per additional year	0.98	0.95–1.01	0.18
**Ongoing therapy[1]**			
Biologics (vs no biologics)	1.24	0.78–1.97	0.36
Immunomodulators (vs no immunomodulators)	1.15	0.69–1.91	0.59
5-ASA (vs no 5-ASA)	0.89	0.56–1.41	0.62

Predictor	aOR	95% CI	p-value
Systemic steroids (vs no steroids)	2.34	0.68–8.05	0.18

aOR, adjusted odds ratio; **CI**, confidence interval; **UC**, ulcerative colitis; **CD**, Crohn's disease; **IBD-U**, inflammatory bowel disease-unclassified; **5-ASA**, 5-aminosalicylic acid.

https://doi.org/10.3390/xxxxx
