# Supplementary material for: Sampling Extension, Chronic Infiltrates, and Eosinophils: Support for the Evaluation of Histological Healing in Inflammatory Bowel Disease with Endoscopic Remission
Source: Diagnostics (Basel). 2026 Mar 2;16(5):739. doi: 10.3390/diagnostics16050739 (PMC12984903; doi:10.3390/diagnostics16050739)
Supplement: Supplementary file 1 [file diagnostics-16-00739-s001.zip › Table S2.pdf]

Table S2. Primary outcome stratified by IBD subtype: histologic activity and sampling extent (1–3 vs >3 sampled segments).

Subtype	Eligible procedures, n	Histologic activity overall, n (%)	1–3 segments: activity, n/N (%)	>3 segments: activity, n/N (%)	p-value	FDR-adjusted p-value (q)
UC	227	73 (32.2)	13/58 (22.4)	60/169 (35.5)	0.07	0.14
CD	104	29 (27.9)	6/29 (20.7)	23/75 (30.7)	0.31	0.31
IBD-U	40	8 (20.0)	2/11 (18.2)	6/29 (20.7)	N/A*	N/A*

* Inferential testing not performed due to limited subgroup size; descriptive estimates provided.
